# The role of deubiquitinases in cardiovascular diseases: mechanisms and therapeutic implications

**DOI:** 10.3389/fcvm.2025.1582049

**Published:** 2025-05-01

**Authors:** Xiangyu Fei, Chao Song, Jian Cui, Yuqing Li, Xiaoyong Lei, Huifang Tang

**Affiliations:** ^1^ School of Pharmacy, Hengyang Medical College, University of South China, Hengyang, Hunan, China; ^2^ Hunan Provincial Key Laboratory of Multi-omics And Artificial Intelligence of Cardiovascular Diseases, University of South China, Hengyang, Hunan, China; ^3^ Department of Cardiology, Hengyang Medical School, The First Affiliated Hospital, University of South China, Hengyang, Hunan, China; ^4^ Clinical Research Center for Myocardial Injury in Hunan Province, The First Affiliated Hospital, Hengyang, Hunan, China; ^5^ Institute of Cardiovascular Disease, Hengyang Medical School, The First Affiliated Hospital, University of South China, Hengyang, Hunan, China

**Keywords:** deubiquitinases, deubiquitination, ubiquitin-proteasome system, signaling pathways, proteostasis, cardiovascular disease

## Abstract

Cardiovascular diseases (CVDs) have become the leading cause of death globally, surpassing infectious diseases and other chronic illnesses. The incidence and mortality rates of CVDs are rising worldwide, posing a key challenge in public health. The ubiquitination system is a vast and complex. It is an important post-translational modification that plays a crucial role in various cellular processes. Deubiquitination is catalyzed by deubiquitinases (DUBs), which remove ubiquitin (Ub) from ubiquitinated proteins, thereby reversing the ubiquitination process. DUBs play an important role in many biological processes, such as DNA repair, cell metabolism, differentiation, epigenetic regulation, and protein stability control. They also participate in the regulation of many signaling pathways associated with the development and progression of CVDs. In this review, we primarily focus on the role of DUBs in various key pathological mechanisms of atherosclerosis (AS), such as foam cell formation, vascular remodeling (VR), endothelial-to-mesenchymal transition (End-MT), and clonal hematopoiesis (CH). In the heart, we summarize the involvement of DUBs in diseases and pathological processes, including heart failure (HF), myocardial infarction (MI), myocardial hypertrophy (MH) and ischemia/reperfusion (I/R) injury. Additionally, we also explore the diabetic cardiomyopathy (DCM) and the use of doxorubicin-induced cardiotoxicity in clinical settings. A comprehensive understanding of deubiquitination may provide new insights for the treatment and drug design of CVDs.

## 1 Introduction

**Figure 1:**
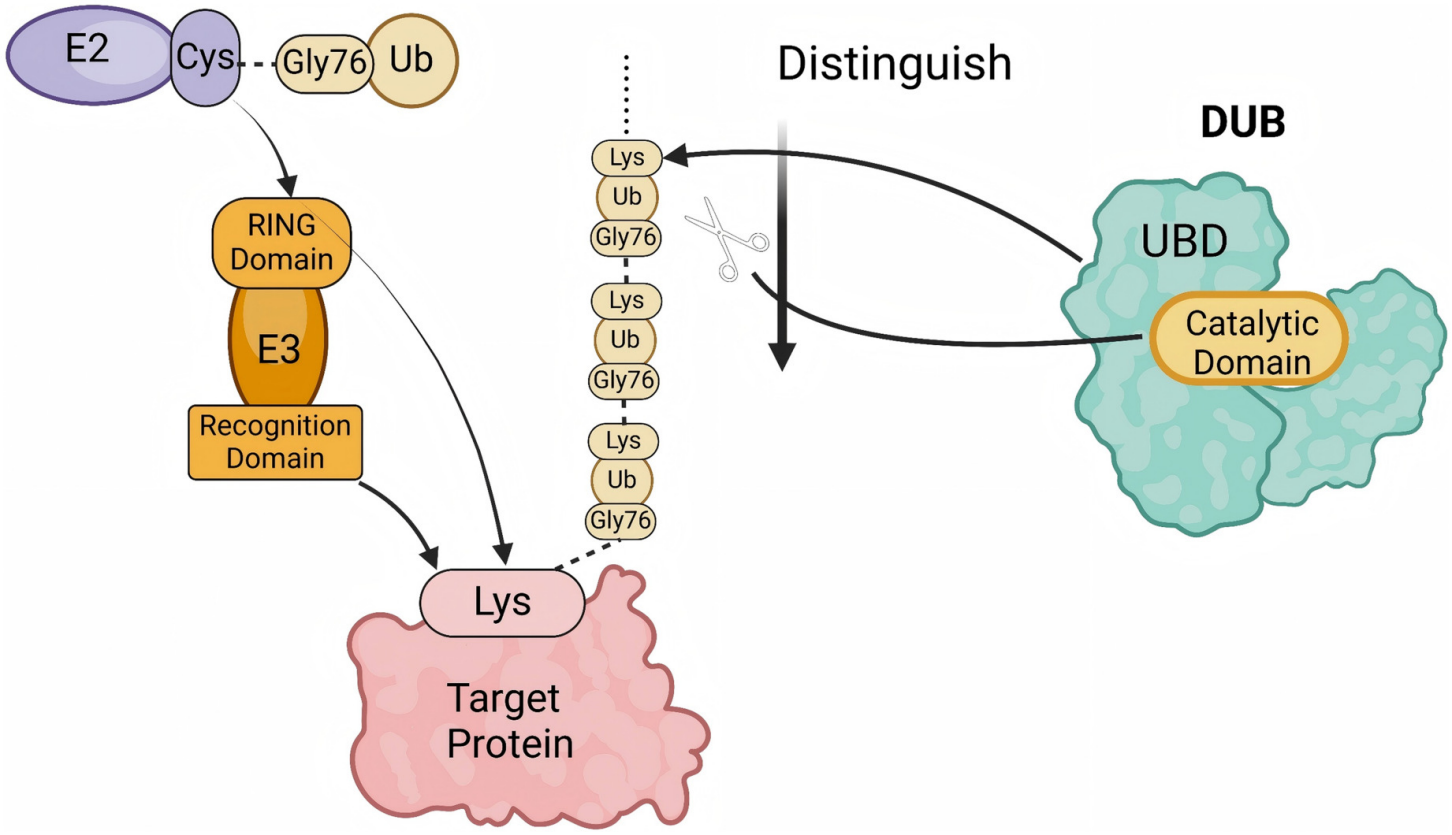
The process of polyubiquitination and deubiquitination. The E2 enzyme carries an activated ubiquitin (Ub) molecule via its cysteine (Cys) residue and transfers it to the target protein under the action of the E3 ubiquitin ligase. The E3 ligase, composed of a RING domain and a recognition domain, facilitates the attachment of ubiquitin to the lysine (Lys) residue of the target protein. Subsequently, a polyubiquitin chain is formed through the linkage between the glycine residue at position 76 (Gly76) of one ubiquitin molecule and the Lys residue of the preceding ubiquitin. Deubiquitinases (DUBs) recognize and cleave ubiquitin chains at specific sites. The ubiquitin-binding domain (UBD) of DUBs identifies the ubiquitin chain, while the catalytic domain performs the cleavage, thereby regulating the ubiquitination levels of proteins. (created with BioRender.com).

Despite the increasing depth of understanding and research into CVDs, their mortality rate remains high (1). The ubiquitin-proteasome system (UPS) plays a significant role in CVDs due to its unique functions. As one of the key protein degradation systems within cells, the UPS is primarily responsible for the degradation and regulation of target proteins. It is involved in the degradation of over 80% of the proteins within cellular proteins (2). The system consists of Ub, ubiquitin-activating enzymes (E1s), ubiquitin-conjugating enzymes (E2s), ubiquitin ligases (E3s), and DUBs. The ubiquitination process, mediated by E1s, E2s, and E3s, is referred to as ubiquitination (3). Protein homeostasis imbalance is a key factor that disrupts cellular homeostasis. These enzymes typically mark their specific target proteins using Ub, leading to their degradation by the proteasome, or further mediate signaling based on the protein's modification sites and the number of attached Ub molecules, thus participating in various physiological processes (4) (Figure 1). The reverse process of ubiquitination is called deubiquitination. Deubiquitination is primarily catalyzed by DUBs, which are classified into seven families based on sequence and structural similarity: ubiquitin-specific proteases (USPs), ovarian tumor proteases (OTUs), ubiquitin c-terminal hydrolases (UCHs), Machado-Josephin domain proteases (MJDs), JAB1/MPN+/MOV34 (JAMM) domain proteases, monocyte chemoattractant protein-Induced protein (MCPIP), and a novel family of DUBs associated with ubiquitin-binding, motif Interacting with Ub-containing novel DUBs (MINDYs) (5). DUBs are a group of proteins that recognize and cleave Ub chains. They interact with substrate proteins, preventing their degradation by the UPS. DUBs contain ubiquitin-binding domains (6), which enable that they perform specific and precise regulation through the recognition and recruitment of ubiquitinated proteins (7). By cleaving the peptide or isopeptide bonds between Ub and its substrate, DUBs remove Ub from ubiquitinated proteins, thereby stabilizing the substrate and reversing ubiquitination (8). Additionally, DUBs regulate signaling pathways to prevent their overactivation, maintaining UPS homeostasis (9). Through Ub removal, DUBs rescue target proteins from degradation signals, preserving their stability (10). The dynamic balance between ubiquitination and deubiquitination is intricately linked to various cellular functions and is critical in disease pathogenesis and therapeutic interventions (11).

**Figure 2:**
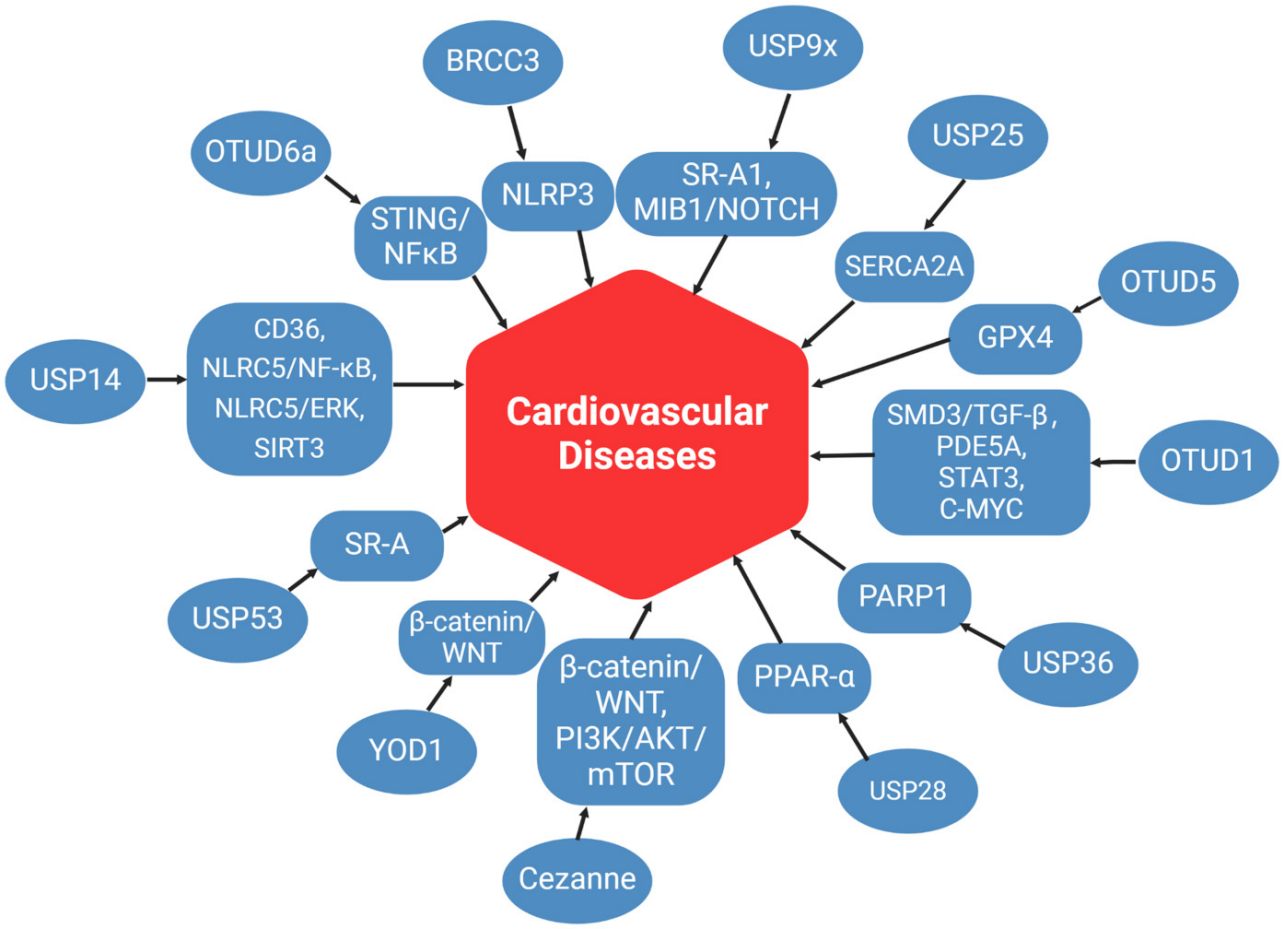
The role of DUBs in CVD. This figure summarizes the mechanisms of DUBs in various pathways associated with CVD, as discussed in this review article. (created with BioRender.com).

To date, more than 100 DUBs have been identified (12). Recent studies have demonstrated that DUBs play crucial regulatory roles in various CVDs and control the onset and progression of disease through multiple mechanisms. This review will systematically examine the role of DUBs in CVDs, categorized by different disease types (Figure 2).

## 2 Atherosclerosis

AS is a multifocal, immune-mediated chronic inflammatory disease that predominantly affects large and medium-sized arteries and is closely associated with lipid deposition. The hallmark pathological feature of AS is excessive cholesterol accumulation in arterial walls, leading to progressive thickening and hardening (13). AS a leading contributor to acute cardiovascular events including MI and stroke, AS represents a complex disease process involving multiple pathophysiological mechanisms such as dysregulated lipid metabolism, aberrant immune responses, and VR (14). uring the onset and progression of AS, DUBs play crucial roles in regulating several processes, including endothelial cell function, immune cell activation, inflammation, and lipid uptake. These findings suggest that DUBs may serve as potential therapeutic targets for AS treatment. We will systematically elucidate the mechanistic role of DUBs in the initiation and progression of atherosclerosis across its different developmental stages.

### 2.1 Endothelial dysfunction and inflammatory regulation

Atherosclerosis can be broadly divided into several stages: lipid deposition, persistent inflammatory response, foam cell formation, vascular smooth muscle cell (VSMC) proliferation, and VR (15). In the early stage of atherosclerosis, no apparent atherosclerotic plaques have formed in the vascular wall; however, a series of pathophysiological changes have already begun to take place. The earliest alterations occur in endothelial cells, with endothelial cell injury being defined as the initiating event of atherosclerosis (16). Endothelial cells, positioned at the interface between solid and semi-solid states, serve as the outermost protective barrier of blood vessels. Under physiological conditions, they maintain vascular homeostasis by regulating vascular tone, inhibiting thrombosis, and modulating inflammatory responses (17). However, when exposed to pathological stimuli such as from disturbed blood flow (18), Oxidative stress (19), inflammatory factors and elevated lipid levels, endothelial cells undergo an inflammatory response, leading to structural alterations and functional impairment, ultimately disrupting vascular homeostasis, by suppressing the inflammatory response to reduce endothelial cell injury, the progression and development of atherosclerosis can be effectively alleviated. A study has shown that USP14 expression is significantly downregulated in atherosclerotic plaques. Further experiments revealed that overexpression of USP14 alleviates ox-LDL-induced endothelial inflammation, primarily by deubiquitinating NLRC5, thereby inhibiting NF-*κ*B activation and reducing the release of pro-inflammatory cytokines (20). Interestingly, another study by Zhang et al. reported that USP14 expression is upregulated in the serum of atherosclerosis patients, and overexpression of USP14 promotes the activation of the Smad2/3 signaling pathway via NLRC5 deubiquitination (21), leading to increased secretion of pro-inflammatory cytokines. These seemingly contradictory findings, however, can be reasonably explained. Firstly, NLRC5 itself exhibits dual functions. While it is generally considered an inhibitor of inflammation, it can also act as a pro-inflammatory regulator under certain conditions (22). Secondly, USP14's deubiquitination activity may result in different biological effects depending on the type of ubiquitin chains removed. Lastly, USP14 exerts distinct functions in different cell types and pathological conditions. For instance, in macrophages, downregulation of USP14 suppresses inflammation (23). In THP-1 and RAW264.7 cells, inhibition of USP14 inactivates the ERK signaling pathway, thereby reducing LPS-induced inflammation (24). In TCMK-1 cells, USP14 inhibition downregulates TFAP2A while upregulating TBK1, leading to reduced oxidative stress and inflammation (25). Undoubtedly, USP14 plays a crucial role in the pathogenesis of atherosclerosis. Although its precise mechanisms require further investigation, current evidence strongly suggests that USP14 is closely linked to inflammation regulation. Specific USP14 inhibitors, such as IU1 and other compounds, have been identified (26). Unfortunately, these inhibitors remain in the experimental stage and have yet to be translated into clinical applications. In the future, USP14 may emerge as a promising therapeutic target for controlling inflammation and treating atherosclerosis, offering new avenues for disease intervention. End-MT is a critical phenotypic alteration associated with endothelial dysfunction (27). Under maladaptive and pathological conditions, newly transformed mesenchymal cells undergo fibrosis, leading to excessive extracellular matrix deposition, increased tissue stiffness, and ultimately impaired vascular function, thereby promoting disease progression. OTUD1, a deubiquitinase belonging to the OTUD family, has been identified as a key regulator of End-MT. Huang et al. Demonstrated (28) that OTUD1 deubiquitinates SMAD3, thereby exacerbating AngII-induced VR and collagen deposition by promoting End-MT. SMAD3 is a crucial signal transduction protein involved in multiple cardiovascular diseases (29–31). As a member of the SMAD family, SMAD3 forms a complex with other family proteins to regulate gene expression (32). OTUD1 stabilizes SMAD3 and enhances the formation of the SMAD3/SMAD4 complex, thereby modulating the transcription of genes associated with End-MT and VR. β-catenin is a central regulatory factor in the Wnt signaling pathway, playing a pivotal role in cellular proliferation and differentiation. It has been extensively investigated as a therapeutic target in cardiovascular and other diseases (33, 34). The deubiquitinase YOD1 interacts with β-catenin via its OTU domain, removing its K48-linked ubiquitin chains, thereby preventing its degradation. This process leads to the aberrant nuclear accumulation of β--catenin, which subsequently enhances the transcription of EndMT-related genes, further promoting endothelial dysfunction, VR, and pathological progression. Collectively, these molecular mechanisms highlight the intricate regulatory network governing End-MT and its role in endothelial dysfunction and VR. The pathological alterations induced by End-MT not only compromise endothelial integrity but also create a pro-inflammatory and pro-oxidative microenvironment within the vasculature. These changes, in turn, facilitate the recruitment and infiltration of immune cells, further amplifying vascular damage and contributing to the progression of atherosclerosis.

### 2.2 Lipid deposition and foam cell formation

Following endothelial cell injury, structural alterations and functional impairment lead to a marked increase in vascular permeability, facilitating the penetration of large amounts of LDL into the subendothelial space. Under the influence of reactive ROS, LDL undergoes oxidation, forming ox-LDL (35). Simultaneously, the expression of ICAM-1 and VCAM-1 is upregulated, further enhancing monocyte adhesion and enabling their transendothelial migration into the intimal layer. Upon stimulation by MCP-1 and TNF-α, monocytes differentiate into macrophages. Macrophages, through scavenger receptors such as SR-A1, CD36, and LOX-1, indiscriminately uptake ox-LDL (36). Since these receptors are not subject to negative feedback regulation by intracellular cholesterol levels, the continuous accumulation of ox-LDL within macrophages results in excessive intracellular lipid overload, ultimately driving their transformation into foam cells (36). Foam cells not only exacerbate the inflammatory response but also significantly increase the risk of plaque rupture. As early as 2006, studies reported that heightened activity of the UPS is closely associated with increased plaque inflammation and vulnerability (37), suggesting that UPS plays a crucial role in the pathogenesis and progression of atherosclerosis. Furthermore, deubiquitination may serve as a key regulatory factor in foam cell formation, warranting further investigation into its underlying mechanisms. In macrophages and mouse models with USP9X deficiency, studies have shown a significant increase in ox-LDL uptake, lipid accumulation, lesion macrophage content, and necrotic core expansion. Mechanistically, USP9X inhibits foam cell formation by removing K63-linked polyubiquitination of SR-A1 at the K27 site (38). Additionally, USP14 has been identified as a deubiquitinase for CD36, promoting foam cell formation by deubiquitinating CD36 in THP-1 and RAW264.7 macrophages (39). Similarly, VSMC serve as another major source of foam cells, and the deubiquitinase USP53 facilitates lipid uptake and accelerates foam cell formation by stabilizing SR-A through deubiquitination (40). Beyond lipid uptake, macrophage polarization plays a critical role in the pathogenesis and progression of atherosclerosis (41). In the early stages of atherosclerosis, M1 macrophages secrete pro-inflammatory cytokines such as TNF-α and IL-6, exacerbating local inflammation and arterial wall damage, thereby accelerating lesion development (42). In contrast, during disease progression, M2 macrophages contribute to lesion repair and plaque stabilization by secreting anti-inflammatory cytokines like IL-10, thus slowing the progression of atherosclerosis Modulating macrophage polarization may offer a novel therapeutic approach for atherosclerosis (43). Studies have demonstrated that the deubiquitinase Mysm1 plays a crucial role in macrophage survival and polarization. Its deficiency leads to accelerated proliferation and increased production of pro-inflammatory factors, promoting an M1-like phenotype (44). In another study, Rui et al. revealed that USP14 stabilizes cGAS, thereby enhancing cGAS-STING pathway activation and promoting ox-LDL/4-HNE-induced pro-inflammatory M1 macrophage polarization. Conversely, inhibiting USP14 facilitates IL-4/IL-13-induced anti-inflammatory M2 macrophage polarization, alleviating inflammation in atherosclerosis (23). However, whether USP14 can directly deubiquitinate cGAS to stabilize the cGAS protein remains unclear.Collectively, DUBs play a pivotal role in regulating macrophage lipid uptake and polarization. Further exploration of their specific mechanisms may contribute to the development of novel therapeutic strategies for atherosclerosis.

### 2.3 VSMC proliferation, migration, and VR

As inflammation persists and foam cells accumulate, structural changes gradually occur in the vascular wall, characterized by VSMC proliferation, migration, and extracellular matrix remodeling. This process, known as vascular remodeling, is a critical hallmark of atherosclerosis progression (45). The phenotypic transition, proliferation, and migration of VSMC not only influence the stability of atherosclerotic plaques but also directly determine the severity of arterial stenosis. Additionally, End-MT and macrophage polarization collectively regulate vascular remodeling, further promoting vascular stiffening and fibrosis, thereby exacerbating pathological vascular remodeling (46). Cezanne is a DUBs belonging to the OTUD family. It was first characterized in 2001 as a negative regulator of the NF-*κ*B signaling pathway (47). Subsequent studies revealed that it can be induced by various pro-inflammatory cytokines and functions as an inhibitor of the NF-κB signaling pathway, thereby forming a negative feedback loop in inflammatory cytokine signaling (48). Recent studies have revealed that the DUBs Cezanne plays a pivotal role in VSMC proliferation, migration, and arterial remodeling. Specifically, Cezanne regulates the Wnt/β-catenin signaling pathway by deubiquitinating β-catenin, thereby modulating the expression of cysteine-rich protein 61. Furthermore, analyses of human atherosclerotic tissue samples have provided additional evidence supporting the role of Cezanne in disease progression (49). The Wnt/β-catenin signaling pathway plays a crucial role in the development and pathological remodeling of the cardiovascular system. Dysregulation of this pathway is closely associated with the onset and progression of various cardiovascular diseases (50). Targeting β-catenin for therapeutic intervention has shown promise in cancer research (51), and is gaining attention for its potential in cardiovascular diseases. However, as an intrinsically disordered protein, β-catenin lacks well-defined drug-binding pockets and exhibits poor metabolic stability, posing significant challenges for direct targeting (52). Consequently, indirect regulatory strategies have emerged as a promising alternative. DUBs play a crucial role in cardiovascular diseases and offer new avenues for intervention. For instance, Cezanne modulates β-catenin stability and signaling by regulating its ubiquitination status. Developing therapeutics targeting upstream DUBs such as Cezanne may overcome the limitations of direct β-catenin targeting and expand treatment strategies for cardiovascular diseases. Notably, USP10 (53) and USP14 (54) have also been implicated in the regulation of VSMC proliferation and migration, highlighting the broader involvement of DUBs in vascular remodeling. DUBs play a critical regulatory role in both vascular remodeling and vascular calcification during atherosclerosis. These discoveries not only enhance our understanding of the pathological mechanisms underlying atherosclerosis but also provide potential therapeutic targets for anti-atherosclerotic and anti-calcification strategies.

### 2.4 Clonal hematopoiesis

In recent years, CH has been recognized as an independent risk factor for cardiovascular diseases, alongside traditional risk factors such as smoking and low-density lipoproteins (55). CH is characterized by the presence of mutant hematopoietic cell clones with selective proliferative advantages in peripheral blood (56). It is closely associated with atherosclerosis, primarily by promoting chronic inflammation and exacerbating vascular injury (57). CH is typically driven by somatic mutations in HSCs, such as TET2, DNMT3A, and ASXL1 mutations, which enhance the pro-inflammatory properties of immune cells, including monocytes and macrophages, thereby intensifying vascular inflammation. Among these mutations, TET2 deficiency promotes NLRP3 inflammasome activation, leading to increased secretion of IL-1β and IL-18, which in turn induces endothelial cell injury, foam cell accumulation, and aberrant proliferation and migration of VSMC, thereby accelerating the initiation and progression of atherosclerotic lesions (58). BRCC3 plays a critical role in CH-driven atherosclerosis. As a DUBs, BRCC3 directly deubiquitinates NLRP3, enhancing its stability and sustaining inflammasome activation. Consequently, aberrant BRCC3 activation amplifies pro-inflammatory signaling, exacerbating vascular inflammation and atherosclerosis (59). Targeting BRCC3 represents a promising strategy to mitigate CH-induced atherosclerosis. Inhibiting BRCC3 prevents NLRP3 deubiquitination, thereby reducing excessive inflammasome activation and suppressing IL-1β secretion, ultimately alleviating vascular inflammation associated with CH. Unlike traditional NLRP3 inhibitors such as MCC950 (60), which block NLRP3 assembly and activation by targeting the NACHT domain and have demonstrated efficacy in reducing atherosclerotic plaque formation in murine models (61), BRCC3 inhibition offers distinct advantages. While MCC950 has shown promising anti-inflammatory effects in various disease models (62, 63), its clinical application has been hindered by hepatotoxicity. Moreover, traditional anti-inflammatory therapies typically target key inflammatory cytokines or signaling pathways directly. While effective in reducing inflammation, they may also compromise baseline immune function, increasing the risk of infections and other adverse effects. In contrast, BRCC3 inhibition provides a more refined regulatory mechanism. By selectively modulating NLRP3 stability, BRCC3 inhibition specifically attenuates excessive inflammasome activation driven by TET2 deficiency while preserving baseline inflammasome function. This targeted approach not only minimizes adverse effects but also maintains essential immune defense mechanisms, positioning BRCC3 inhibition as a safer and more precise therapeutic strategy compared to conventional anti-inflammatory treatments.

## 3 Heart failure

HF is a clinical syndrome characterized by the heart's inability to pump blood effectively to meet the body's metabolic demands or maintain normal blood flow. The causes of HF are diverse, typically involving structural and functional changes in the heart, along with the interactions of systemic factors. Additionally, DUBs play a crucial regulatory role in the onset and progression of HF. The following discussion will explore the mechanisms through which several DUBs contribute to this process.

### 3.1 Myocardial hypertrophy

The primary function of the heart is to ensure adequate perfusion of peripheral organs, meeting their metabolic demands under both normal and stressed conditions. To accomplish this under increased preload or afterload, the heart and individual cardiomyocytes typically undergo hypertrophy. This hypertrophic response initially develops as an adaptive mechanism to both physiological and pathological stimuli. However, pathological hypertrophy often progresses to HF over time (64). Each form of hypertrophy is initiated and regulated by a variety of cellular signaling pathways. Over the past decade, many previously unrecognized mechanisms have been identified that regulate MH from both positive and negative aspects, including cellular metabolic proliferation (65, 66), immune responses (67, 68), and epigenetic modifications (69). Currently, classic medications, including β-adrenergic blockers and renin-angiotensin-aldosterone system inhibitors, are used to improve pathological ventricular hypertrophy. However, the incidence of HF remains high, suggesting that other signaling pathways or regulatory proteins also play crucial roles in pathological hypertrophy.

Calcium ion (Ca^2+^) cycling plays a crucial role in the contraction and relaxation of cardiomyocytes. The sarcoplasmic reticulum (SR), as the organelle that stores Ca^2+^, is primarily responsible for mediating the uptake and release of Ca^2+^ during the contraction-relaxation process. Dysregulation of sarcoplasmic reticulum function can severely impair Ca^2+^ cycling, leading to abnormalities in cardiomyocyte function (70). Sarcoplasmic/endoplasmic reticulum Calcium ATPase 2a (SERCA2a) is the cardiac-specific SERCA isoform, responsible for mediating the re-uptake of Ca^2+^ into the SR during cardiomyocyte diastole. SERCA2a regulates cardiac contractility and relaxation by controlling Ca^2+^ uptake, thus influencing cardiac function. In HF, the expression and activity of SERCA2a are reduced. Gene therapy aimed at increasing SERCA2a expression in the heart has been shown to be effective (71). USP25 is a product of the 21q11.2 gene, consisting of 25 exons, and can produce three isoforms through alternative splicing: USP25a, USP25b, and USP25m. USP25m is specifically expressed in skeletal muscle and the heart, and it is upregulated during myogenesis, suggesting a potential role for USP25 in cardiac biology. Studies have shown that the deletion of USP25 exacerbates MH and cardiac dysfunction induced by Ang II and TAC. In contrast, restoring USP25 expression significantly improves pathological cardiac hypertrophy. Mechanistically, USP25 regulates the uptake of Ca^2+^ by deubiquitinating and stabilizing SERCA2a via K48-linked deubiquitination (72), thereby influencing cardiac function.Another member of the OTUD family, OTUD6a, has also been found to be involved in the regulation of MH (73). It primarily regulates the STING-NF-κB inflammator*y* axis by deubiquitinating the K48-linked Ub chains on STING, thereby maintaining STING stability. Inhibiting OTUD6a significantly reduces the activation of the STING signaling pathway, thus slowing down the progression of MH. Targeting DUBs to regulate MY could become a new direction for the treatment of HF.

### 3.2 Myocardial infarction

MI is a severe cardiovascular event characterized by ischemic necrosis of cardiomyocytes due to a sudden reduction or complete cessation of coronary blood flow. The underlying pathophysiological mechanisms primarily involve atherosclerotic plaque rupture, subsequent thrombus formation, and coronary artery spasm, ultimately leading to myocardial perfusion impairment and irreversible cardiomyocyte injury. The occurrence of MI can trigger a range of severe cardiovascular complications, including HF (74). OTUD1 plays a crucial role not only in VR but also in cardiac diseases. Wang et al. (75) established MI and HF mouse models by performing MI surgery and administering isoproterenol, respectively, to investigate the role of OTUD1. The study found that the expression levels of OTUD1 were significantly elevated in both models. Knockdown of OTUD1 resulted in substantial structural improvements, alleviation of cardiac damage, and restoration of cardiac function. The mechanism primarily involves OTUD1 deubiquitinating phosphodiesterase 5A (PDE5A) in cardiomyocytes, inhibiting its degradation, and subsequently suppressing the activation of the cGMP-PKG pathway. This process may be a key factor leading to the disruption of calcium homeostasis and MH. cGMP and its kinase PKG play critical roles in regulating cardiomyocytes and cardiac function (76). PDE5A, a member of the phosphodiesterase family, plays an important regulatory role in the cardiovascular system (77). Numerous studies have demonstrated that inhibiting PDE5A can effectively alleviate the progression of HF (78, 79). Therefore, investigating the upstream regulatory proteins of PDE5A and their underlying mechanisms holds significant clinical implications for the treatment of HF. Additionally, another study revealed that OTUD1 can regulate pathological cardiac remodeling and HF by removing K63-linked ubiquitination from STAT3, thereby promoting phosphorylation at the Y705 site, increasing p-STAT3 levels, and facilitating its nuclear translocation (80). Liu et al. identified that OTUD5, another member of the OTUD family closely related to OTUD1, acts as a deubiquitinase for glutathione peroxidase 4 (GPX4) in cardiomyocytes (81), 4-hydroxy-2-nonenal (4-HNE), a reactive aldehyde derived from lipid peroxidation, is commonly produced during oxidative stress. Numerous studies have confirmed that 4-HNE is significantly elevated during MI and is a primary contributor to cell death (82, 83). In this study (81), the authors demonstrated that 4-HNE induces carbonylation of GPX4, impairing its normal function and promoting K48-linked ubiquitination, which leads to proteasomal degradation and triggers ferroptosis in cardiomyocytes. OTUD5 counteracts this process by removing Ub chains from GPX4, thereby protecting it from 4-HNE-mediated damage and degradation. This mechanism effectively prevents ferroptosis and mitigates ischemia-reperfusion-induced cardiac injury (84), Preventing cardiomyocyte damage represents a critical strategy in treating cardiac diseases. As a deubiquitinase for GPX4, OTUD5 plays a vital role in maintaining cellular homeostasis. Its depletion has been shown to exacerbate ferroptosis, evidenced by a significant reduction in the SLC7A11/SLC3A2 complex in OTUD5-silenced cardiomyocytes. This suggests that OTUD5 may also regulate ferroptosis through transcriptional pathways, providing a broader understanding of its cardioprotective mechanisms.Targeting OTUD5 in clinical practice could represent a promising therapeutic strategy for patients undergoing reperfusion therapy after MI.

In CVDs, DUBs critically regulate cardiomyocyte stability and function. They contribute to HF pathogenesis by modulating pathological MH, post-myocardial infarction remodeling, and maladaptive responses. DUBs mediate cardiac injury and remodeling through transcriptional regulation, inflammatory signaling, and cell survival mechanisms. Current research provides novel insights into HF progression mechanisms and identifies DUBs as potential therapeutic targets.

## 4 Cardiomyopathy

Based on etiology and pathophysiological characteristics, cardiomyopathy can be classified into genetic and acquired types, including dilated cardiomyopathy, hypertrophic cardiomyopathy, restrictive cardiomyopathy, and secondary cardiomyopathy caused by specific underlying conditions (85). The main features of cardiomyopathy include myocardial structural remodeling, ventricular dysfunction, and the potential progression to HF and arrhythmias (86). Among acquired cardiomyopathies, toxic and metabolic factors can significantly affect myocardial function. Doxorubicin (DOX), due to its potent anticancer properties, is widely used in clinical practice. However, its severe cardiotoxicity limits the clinical doses that can be administered. DOX-induced cardiotoxicity is a common form of drug-induced cardiomyopathy in clinical practice, primarily characterized by oxidative stress injury in cardiomyocytes, mitochondrial dysfunction, and impaired cardiac contractility. Therefore, studying its toxic mechanisms and developing targeted prevention or treatment strategies are currently key focuses of both clinical and basic research (87). OTUD1 has been found to deubiquitinate c-MYC, and inhibiting OTUD1 can effectively protect mice from DOX-induced cardiac dysfunction (88). Moreover, DUBs such as USP36 (89), USP14 (90), and Cezanne (91) play significant roles in DOX-induced cardiac dysfunction. Although their specific targets and the signaling pathways they modulate differ, the cellular processes ultimately regulated by these DUBs, including apoptosis and oxidative stress, are closely associated with their toxic effects. These mechanisms are key contributors to the cardiac toxicity observed (92). Targeting DUBs to mitigate the cardiac toxicity they induce offers a promising new direction for research and potential therapeutic strategies.

In addition to toxic factors, metabolic abnormalities also play a crucial role in the development of cardiomyopathy. DCM is a specific form of cardiomyopathy caused by diabetes, characterized by cardiomyocyte hypertrophy, fibrosis, cardiac dysfunction, and arrhythmias (93). DUBs play a crucial role in DCM. In the db/db mouse model of diabetes, differences in UPS expression in diabetic myocardial tissue were already observed in young db/db mice, with a significant reduction in the levels of certain DUBs (94). Recent studies have indicated that the primary cause of HF in DCM is the impairment of cardiomyocyte function. Mitochondrial dysfunction is a critical and often overlooked aspect of the pathology and pathogenesis of DCM. Xie et al. Discovered (95), that the expression of USP28 was significantly reduced in the hearts of diabetic patients and db/db mice. Furthermore, compared to the control group, Myh6-Cre+/USP28fl/fl mice showed more severe, progressive heart dysfunction, lipid accumulation, and mitochondrial disruption. Further investigation revealed that USP28 stabilizes peroxisome proliferator-activated receptor *α* through deubiquitination, which in turn promotes the transcription of mitofusin 2, thus protecting mitochondrial morphology and function and alleviating diabetic HF. Protein C (PC), a natural anticoagulant, is activated to form activated protein C (aPC), which has been found to induce the key deubiquitinase OTUB1 through PAR1 and EPCR signaling pathways. This induction helps maintain the expression of YB-1. The sustained levels of YB-1 inhibit the transcription of MEF2B, thereby protecting the heart from the effects of DCM (96). It is worth noting that Xigris, a drug approved in 2001 for the treatment of sepsis, contains the active ingredient recombinant activated protein C (rAPC). However, it was withdrawn by the FDA due to severe bleeding complications. Recent studies, however, have indicated that aPC plays an important role in I/R injury as well (97). In conclusion, although research on the role of DUBs in DCM is still limited, it is undeniable that targeting DUBs offers a novel approach for treating DCM.

## 5 Conclusions

The process of deubiquitination plays a critical regulatory role in the onset and progression of CVDs, particularly in regulating key signaling pathways and maintaining cellular homeostasis (Table 1). Clinically, the application of DUBs is primarily focused on cancer, neurodegenerative diseases, inflammation, and immunity. These enzymes mainly regulate protein homeostasis, the cell cycle, DNA repair, and signal transduction, thus participating in many key biological processes. In cancer treatment, deubiquitinase inhibition strategies can generally be divided into two types: stabilization of the enzymatic active site conformation and allosteric inhibition of non-catalytic sites (101). Many newly developed DUB inhibitors, such as Novartis's CSN5i-3 (102) or the already marketed drug pimozide (103), have been found to exhibit significant functions in the field of cancer therapy.

**Table 1:** Deubiquitinases are involved in cardiovascular pathways.

DUBs	Deubiquitinated Substrates	Signaling pathway	Pathological Process	Reference
USP9x	SR-A1		Inflammation, Formation of foam cells	(38)
	MIB1	NOTCH	Vascular calcification	(98)
USP53	SR-A		Formation of foam cells	(40)
USP14	CD36		Formation of foam cells	(39)
	NLRC5	NF-κB	Inflammation	(20)
	NLRC5		Endothelial-mesenchymal transition	(21)
	NLRC5	ERK	Inflammation	(24)
	SIRT3		Oxidative stress	(90)
BRCC3	NLRP3		Clonal hematopoiesis	(99)
YOD1	β-catenin	WNT	Endothelial-mesenchymal transition,Vascular remodeling	(100)
Cezanne	β-catenin	WNT	Vascular remodeling	(49)
	PI3K	AKT/mTOR	Doxorubicin-induced cardiomyopathy	(91)
USP25	SERCA2A		Myocardial hypertrophy	(72)
OTUD6a	STING	STING/NF-κB	Myocardial hypertrophy	(73)
OTUD1	SMAD3	TGF-β	Endothelial-mesenchymal transition	(28)
	PDE5A		Myocardial hypertrophy	(75)
	STAT3		Heart failure	(80)
	C-MYC		Oxidative stress	(88)
OTUD5	GPX4		Ischemia-reperfusion injury	(81)
USP36	PARP1		Oxidative stress	(89)
USP28	PPAR-α		Heart failure	(95)

In cardiovascular research, there has been significant interest in the development of DUB-targeting drugs, but most of these studies remain in the predictive or experimental stages, with no drugs successfully translated for clinical use. Targeting DUBs offers significant advantages over traditional small molecule drugs. Instead of merely inhibiting the active sites of proteins and reducing their activity, DUB inhibitors can completely degrade the target proteins, effectively eliminating the residual activity that often leads to side effects with traditional drugs. Although the role of DUBs in CVDs is gradually being revealed, the specific mechanisms remain incompletely understood. Future research should focus on exploring the relationships between DUBs, signaling pathways, transcription factors, and other biomarkers relevant to CVDs, to further investigate their multifaceted roles in disease progression.
